# Transforming Standard of Care for Spine Surgery: Integration of an Online Single-Session Behavioral Pain Management Class for Perioperative Optimization

**DOI:** 10.3389/fpain.2022.856252

**Published:** 2022-05-02

**Authors:** Sara A. Davin, Jason Savage, Nicholas R. Thompson, Andrew Schuster, Beth D. Darnall

**Affiliations:** ^1^Neurological Institute, Center for Spine Health, Cleveland Clinic, Cleveland, OH, United States; ^2^Cleveland Clinic, Department of Quantitative Health Science, Neurological Institute, Cleveland, OH, United States; ^3^Department of Anesthesiology, Perioperative and Pain Medicine, Stanford University School of Medicine, Palo Alto, CA, United States

**Keywords:** behavioral medicine, chronic pain, brief intervention, spine surgery, pain medicine, perioperative, psychology, Empowered Relief

## Abstract

Estimates suggest that 10-40% of lumbar spine surgery patients experience persistent post-surgical pain (PPSP). PPSP is associated with 50% greater healthcare costs, along with risks of emotional distress and impaired quality of life. In 2019, U.S. Health and Human Services identified brief and digital behavioral treatments as important for pain management after surgery. Indeed, brief behavioral pain treatments delivered in the perioperative period may offer patients a low burden opportunity to acquire essential pain coping strategies for enhanced surgical recovery. Additionally, the COVID-19 pandemic has diminished in-person pain treatment access during extended perioperative time frames, thus underscoring the need for on-line options and home based care. This report describes the integration of an online, live-instructor delivered single-session pain self-management intervention (Empowered Relief) into the standard of care for lumbar spine surgery. Here, we apply the RE-AIM framework; describe systems implementation of the Empowered Relief intervention in a large, academic medical center during the COVID-19 pandemic; describe operational challenges and financial considerations; and present patient engagement data. Finally, we discuss the scalable potential of Empowered Relief and other single-session interventions in surgical populations, their importance during extended perioperative periods, practical and scientific limitations, and new directions for future research on this topic.

## Introduction

Global estimates suggest that close to 313 million surgeries are performed annually (1) with approximately 500,000 lumbar spine surgeries each year in the United States (2). Up to 80% of spine surgery patients experience post-surgery discomfort (3) and 20% will experience persistent post-surgical pain (PPSP). PPSP is associated with greater healthcare costs up to 10 years post-surgery (4), greater depression, and increased risks for medical complications due to prolonged use of non-steroidal anti-inflammatory drugs or prescription opioids (5). The perioperative period is a critical time to apply non-pharmacological interventions that target pre-surgery anxiety, help patients set reasonable expectations for surgery, and support lifestyle behaviors that facilitate recovery after surgery, including adaptive skills to self-manage acute post-operative pain.

The COVID-19 pandemic has created major symptom challenges for patients seeking pain care and spine surgery, including exacerbated pain, increased distress, and decreased health promoting behaviors, such as physical activity (6). Prolonged periods of social distancing during the pandemic, increased isolation and loneliness present additional risks for increased pain (7). Disruption in care, sometimes including months-long surgical delays, have left patients with few options to manage increased pain, distress, and anxiety within the context of an extended pre-surgical timeframe. One report documented up to one-third of delayed elective spine surgeries remained rescheduled 8 months later, with fears of COVID-19 exposure cited as the primary reason for surgical deferment (8). Given pre-existing patient need for integrated perioperative care coupled with COVID-related surgical delays, we underscore the importance of accessible (e.g., online) and low burden perioperative pain interventions.

Few studies have examined the efficacy of behavioral pain treatments in the perioperative period, and even fewer have examined brief and online treatment delivery. Behavioral or “psychological” treatments for pain typically include pain education, strategies to reduce unhelpful ways of interpreting pain, and modification of disabling behaviors. Behavioral therapies for pain consistently demonstrate improvements in pain and distress compared to those that receive standard health education or exercise programs (9). A recent systematic review and meta-analysis of 15 randomized controlled trials reported that education (provision of information and support) in the perioperative period was ineffective, whereas there was moderate quality evidence for reductions in post-operative pain and physical impairment for active skills-based psychological interventions (10). However, barriers to care access exist due to logistical barriers, infeasibility within the perioperative pathway, and patient treatment burdens. In 2019, the U.S. Health and Human Services (HHS) Pain Management Best Practices Interagency Pain Task Report identified brief and online behavioral treatments as important for scaling behavioral pain care to patients who might otherwise be provided pharmacologic analgesia alone. This HHS report also cited, My Surgical Success and Empowered Relief, two specific brief and online interventions that are relevant to the current report (11).

Empowered Relief is a single-session 2-h pain relief skills class for acute/surgical and chronic pain that was developed by Beth Darnall at Stanford University. Multiple randomized trials provide efficacy evidence for Empowered Relief delivered across different patient populations and delivery formats. Results for a 3-arm randomized controlled trial conducted in 263 adults with chronic low back pain revealed that in-person delivered Empowered Relief was non-inferior to 16-h of CBT for reducing pain catastrophizing, pain intensity, pain interference, and multiple secondary outcomes at 3 months post-treatment (12). A second randomized trial compared online-delivered Empowered Relief to a usual care control group in patients with mixed-etiology chronic pain. Results revealed excellent patient engagement with online Empowered Relief, and clinically meaningful reductions in pain intensity, pain catastrophizing, sleep disturbance, and pain bothersomeness at 3 months post-treatment (13). Empowered Relief was adapted to surgical populations with an online and fully-automated version that meets their need for asynchronous learning (called My Surgical Success; MSS).

Two randomized controlled trials of MSS provide efficacy evidence in breast cancer surgery and orthopedic trauma surgery patients. Results for the first trial in breast cancer surgery revealed that compared to an automated, online health education intervention, within a 14-day window of post-surgical observation of opioid use, MSS reduced time to opioid cessation by 6.5 days (14). MSS demonstrated feasibility with 56% of patients engaging with the online platform, a rate that is substantially higher than many e-health interventions. Additionally patient acceptability met or exceeded the 80% threshold on items related to patient understanding and satisfaction. Results suggested that the brief may be a low-cost and accessible strategy to enhance outcomes in breast cancer surgery.

The second randomized trial of MSS vs. digital health education was conducted in 84 patients undergoing orthopedic trauma surgery. The majority of patients received their assigned digital intervention in the hospital after surgery. Patients assigned to MSS had significantly reduced pain after surgery compared to the health education control group (Ziadni et al., under review)^1^.

The two trials of automated Empowered Relief provide promising early evidence for improving outcomes in different surgical populations. Based upon these findings, in 2019-2020 we implemented at Cleveland Clinic Spine Surgery an uncontrolled feasibility implementation project of automated Empowered Relief in spine surgery patients (Davin and Darnall, unpublished data 2020). The Cleveland Clinic Institutional Review Board assigned the project exempt status; data included patient engagement rates and frequencies related to a treatment satisfaction survey. All spine surgery patients were invited to receive the online treatment and neither compensation nor incentives were provided for treatment engagement or survey completion. Of the 158 patients who expressed interest in receiving the online treatment during an 8-week perioperative window, 58 completed the online treatment (37% treatment engagement rate which was notably lower than for the breast cancer and orthopedic trauma studies). Additionally, 36% (*n* = 21) completed a post-class acceptability and satisfaction survey. Data exist only for the patients who engaged in the treatment and completed a post treatment survey. As such a major selection bias should be considered when interpreting the ratings. Among the surveys collected acceptability was high with 95% of participants rating the treatment as understandable, 85% rated it useful and relevant for the future, and the overall treatment satisfaction rating was 90%.

While automated treatments offer patients great convenience for asynchronous learning, they do not offer scheduled and structured guidance. Moreover, they lack the therapist contact that may be crucial for enhanced patient engagement. In an effort to boost patient engagement in behavioral perioperative pain care, we pivoted from the automated digital version of Empowered Relief (MSS) to an online, live clinician-delivered 2 h Empowered Relief class that was tailored to the spine surgery patient. The primary aim of this report is to describe the pathway that was built to operationalize online Empowered Relief as standard intervention in spine surgery patient care. We present patient engagement rates that support the feasibility of online live-instructor delivered Empowered Relief in spine surgery. Of note, we found no published trials that have investigated either efficacy, feasibility, or clinical implementation of an online, live instructor-delivered pain relief skills intervention in spine surgery.

### Integration in a Large Academic Medical Center: Systems and Process

The Cleveland Clinic is a large multi-specialty academic medical center serving patients locally, across the nation and internationally. The Cleveland Clinic Center for Spine Health serves thousands of patients each year, treating the most complex spine issues. The center's team is comprised of specialists including neurosurgery, orthopedics, medical spine, chronic pain and pain psychology. The Center for Spine Health performs ~2,000 spine surgeries each year.

The clinical implementation of Empowered Relief as standard of care for all chronic pain patients in our center was launched in conjunction with the onset of the COVID-19 pandemic. As part of a quality improvement project, we analyzed participation rates and patient satisfaction with the class based on a post-class e-survey, launched in November 2021. We also wanted to determine if participation in the online Empowered Relief class improved patient-reported outcomes (PROs), specifically pain interference and pain catastrophizing, compared to patients who canceled/no showed. The Cleveland Clinic IRB assigned this project exempt status. Analysis of a 12-month period from January 2021-December 2021 demonstrated a 72% engagement rate in the online class. For the patients who completed Empowered Relief, within-group pre-post changes were statistically significant for the Pain Interference T-score (mean change −1.7, *p* = 0.0007), although did not meet the threshold for minimally important difference (12). Clinical meaningful improvements defined per prior methods (15) in the PCS scores were found among the Empowered Relief completers (mean change −6.1; *p* < 0.0001; moderate effect size [Cohen's D = −0.64]; mean percent improvement of 26.2%). Among patients who canceled or were no-shows, none of the within-group changes were statistically significant (all *P* > 0.27). There were no statistically significant between-group differences in change score, which was attributed to small sample sizes of PROS completion between 1 and 90 days of the class (all *P* > 0.25). Further analyses revealed that individuals in the no show/cancel group were also less likely to follow up with other behavioral appointments, thus further limiting their ability to complete PROs (16). While considering selection bias and lack of control for other treatment effects these preliminary indicators of clinical improvements along with an exceptionally high engagement rate provided support for expansion of Empowered Relief to other populations in our center.

Next, our team created a pathway to operationalize Empowered Relief as standard care for spine surgery patients. Steps to build this clinical pathway were focused on three primary areas: 1) education of providers and patients, 2) efficient and low burden clinical implementation, and 3) continuous improvement of the intervention's integration into standard of care. See Table 1 for further illustration of stakeholder engagement and the process to clinical implementation.

**Table 1:** Stakeholder needs and engagement and alignment with RE-AIM dimensions.

**Stakeholder needs and steps to address them**	
**Patient need (REACH)**	**Implementation method to address the need**
Skills-based pain management during COVID with an extended perioperative time frame	• Clinician delivery of intervention ° Low patient engagement with automated treatment supports a model for therapist-delivery for a single-session intervention (Empowered Relief) • On-line delivery of intervention • A live instructor delivered the class to supports patient convenience and safety during pandemic • Brief and accessible intervention to target the need for additional patient support and continuity of care during an extended perioperative period in the COVID-19 pandemic
**Clinician need (ADOPTION)**	**Implementation method to address the need**
Efficient scheduling and low burden workflow	• Class order through the electronic health record created for rapid scheduling and tracking of patients • Accessible educational materials for patients to support clinicians in communicating the purpose of the class and minimizing additional questions • Use of electronic technology to communicate with patients ° Automated class reminders and login instructions and billing information sent to patients prior to the class
**Approaches to enhancing stakeholder engagement in empowered relief**	
Patients (REACH, ADOPTION)	• Surgeons lead messaging to patient that class will support effective surgical pain management • Online treatment delivery expands access to home-based behavioral pain care during COVID restrictions • Online classes offered 1-2 time weekly • Integrating Empowered Relief as standard care minimizes stigma and assures consistency in the messaging patients receive from multiple care providers
Clinicians (IMPLEMENTATION, MAINTENANCE)	• Identify clinical champions (surgeons, nursing, behavioral medicine, program manager) • Create multi-disciplinary work group • Provide surgical team education on Empowered Relief and operational systems designed to decrease staff burden • Empowered Relief instructors are supported by an administrative staff who attends to participants logistical and tech questions, thereby freeing them to focus on delivering the clinical content • Continuous education to entire spine staff on the class, including updates on referral process, continuous improvement efforts and outcomes

## Framework to Guide Implementation: RE-AIM

The RE-AIM framework, (17) one of the most well-known public health models for evaluating interventions for chronic illness, provides useful guidance in the implementation of Empowered Relief in spine surgery. The five dimensions of the model (Reach, Effectiveness, Adoption, Implementation, and Maintenance) address the multi-faceted components of intervention development, efficacy and ongoing evaluation. RE-AIM has been used widely across a variety of health conditions, including chronic pain (18) and is known for addressing the broad array of factors that may influence an intervention in real-world settings. While all five dimensions apply to our project, the dimensions of Reach, Adoption and Implementation were of central importance in the initial phase of development.

### REACH: Patient Engagement

The RE-AIM model defines reach as the number of individuals in the targeted intervention that are willing to engage in the program. Education of patients and providers was essential to enhance class engagement and project success. The messaging of Empowered Relief for spine surgery as “best practice and standard of care,” in our health system is supported by the previously noted HHS recommendations and designed to enhance receptivity to surgical patients. Additionally, the brief and online format of the class specifically was designed to improve overall access to the intervention.

### Effectiveness

Effectiveness refers to the impact of Empowered Relief class on key outcomes. In the early phases of the project, our team's focus was on feasibility, engagement and acceptability of the intervention. Continued data collection from this project will allow our team to assess class effectiveness on sub-acute pain (3 months post-surgery) and CPSP (6+ months post-surgery), disability and pain catastrophizing, compared to individuals who do not attend/engage in Empowered Relief.

### Adoption

#### Provider Engagement

From a systems standpoint, the RE-AIM dimension of adoption centers upon the enhancement of organizational support (17). Thus, in the development phase of our project the focus was on engaging relevant stakeholders within the Center for Spine Health. To target provider engagement, we created a core group of clinical champions in pain psychology, spine surgery and nursing to support the launch of Empowered Relief for spine surgery patients. A multi-disciplinary work group was created to identify the most efficient workflow and to develop educational materials. In the initial phase of program development the workgroup met on a bimonthly basis.

Our team supported several clinicians certification as Empowered Relief instructors (two psychologists, one advanced nurse practitioner, and one registered nurse). The training for Empowered Relief spans over two full days and includes expert led didactic content, a demonstration patient class, interaction with peers and faculty, and small group skills practice.

The Center for Spine Health received education about the class during monthly staff meetings. In-services were offered with nursing to support roll-out of the project and to enhance stakeholder buy-in. The clinical champions in nursing, surgery and psychology elicited feedback from their team each week on 1) efficiency of the referral process, 2) patient engagement and 3) barriers to implementation. Findings in these three key areas were communicated back to the core workgroup each week.

#### Workflow for Efficient and Low Burden Clinical Implementation

An important factor to facilitate adoption of Empowered Relief as part of standard of care for spine surgery was creation of a workflow that was efficient and low burden.

1. A physician and a nurse champion were identified to educate clinicians in their areas about the class, to enhance clinician engagement, to serve as key stakeholders to identify systems problems.
2. An order for the electronic medical record was developed for the class that allowed real-time scheduling and provider tracking of referral and patient participation.
3. Educational materials were created and distributed to patients at point of scheduling to provide a brief overview of the class and to remind providers to market the program.
4. Additional educational materials were deployed to patients electronically including information on: utilizing the virtual technology, billing/insurance, and a frequently asked questions sheet; this communication helped decrease staff burden in responding to patient inquiries.

### Implementation

The RE-AIM framework refers to implementation as patients' engagement and utilization of the intervention (individual level) and the fidelity of the intervention and adaptations made (systems level).

#### Clinical Implementation in Spine Surgery Patients

All male and female patients aged > 18 years undergoing thoracic or lumbar laminectomies with or without fusion were referred to Empowered Relief prior to surgery. The Institutional Review Board at the Cleveland Clinic assigned this clinical improvement project exempt status. The first Empowered Relief class for the surgical cohort was offered July 1, 2021. Data on participation rates were gathered from July 1, 2021 to March 2, 2022.

The Empowered Relief class recruitment occurred at the time in which spine surgery was recommended by the surgeon. Empowered Relief classes were offered 1-2 times per week to provide flexibility and accommodate patients' schedules with other perioperative appointments. For insurance billing, Health and Behavior Assessment and Intervention Services were used. These codes are designed to address psychological, behavioral, emotional, cognitive and interpersonal factors impacting the treatment of an individual with a diagnosed physical health problem. These codes may also be used to provide tele-therapy services (18).

Online Empowered Relief is delivered by a certified instructor. Our perioperative program limited the size each class cohort to 12 patients. Classes occur through a secure online platform that is integrated with patients' electronic medical record. Family members are also encouraged to participate.

#### Methods

Patient characteristics were summarized using mean and standard deviation for continuous variables and counts and percentages for categorical variables in the full patient sample and stratified by completion of session vs. cancel/no-show. Comparisons were made using two-sample *t*-tests for continuous variables and Fisher's exact test for categorical variables.

#### Patient Satisfaction Survey

Immediately after the class, participants received an e-survey to assess their perceptions about the class, including satisfaction, perceived usefulness of information, and likelihood to use the skills learned; all items use an 10-point scale, where 1 is the lowest rating and 10 is the highest possible rating. This survey has been used in prior work (14). Participants received one e-reminder message to complete the class survey.

#### Results: Patient Engagement in Perioperative Empowered Relief

Of the 78 patients who were scheduled for Empowered Relief from July 2022 through March 2022, 48 attended the class (61.5% engagement rate). Class participant mean age was 62.9 (SD = 12.4) with 45.8% female and 87.5% white. Patients who completed the class were less likely to be female than patients who canceled or no-showed (45.8 vs. 73.3%, P = 0.020). Fifteen patients completed the post-class survey (31.3%), which was launched mid-way through the project (November 2021), thus likely also contributing to small sample size. With the consideration of the small sample size and selection bias, those that did complete the post-class survey reported high satisfaction with the class, with the highest percentage of patients (46.7%) answering a 10/10 (highest value possible) to the question, “How likely are you to use the skills and information you learned?” See Table 2 for descriptive statistics of responses to the patient satisfaction survey.

**Table 2:** Responses from the 15 patients who completed the satisfaction survey (mean and standard deviation, median and interquartile range, and the number and percent of patients who answered 10 for the six numeric scale questions).

	**Mean (SD)**	**Median (IQR)**	**Answered 10 *N* (%)**
How satisfied were you with the class?	8.60 (1.18)	8 (8, 10)	5 (33.3%)
Please rate your likelihood to recommend this class to another person who has chronic pain.	8.47 (2.26)	9 (8.5, 10)	6 (40.0%)
How relevant was this class to you?	8.27 (1.79)	9 (7.5, 10)	5 (33.3%)
How useful was the information presented in the class?	8.47 (1.41)	9 (8, 9.5)	4 (26.7%)
How likely are you to use the skills and information you learned?	8.93 (1.49)	9 (9, 10)	7 (46.7%)
How likely are you to participate in additional pain psychology services or programs as a result of participation in the class?	7.60 (1.72)	8 (6.5, 9)	2 (13.3%)

### Maintenance: Continuous Improvement of ER Integration Into Standard of Care

The RE-AIM maintenance dimension refers to 1) durability of the intervention on an individual level and 2) the larger systems and organizational level buy in to sustain continuation of the project. Important challenges were identified during clinical implementation. These valuable learning points were used to continually improve the process for our team and our patients targeting both the systems and individual level.

#### Integration of the Class Into a Demanding Pre-operative Period

The pre-operative period is a busy and stressful period for the surgical candidate. Integration of the class into the other pre-operative requirements posed a challenge; to enhance patient engagement the class was strongly encouraged by all clinicians, but not mandated for surgery. Our administrative staff pro-actively scheduled the patient into the class to decrease burden on the patient or nursing staff. The nursing team expressed concerns upon the project launch for the potential to increase burden on their workload. Steps were taken to minimize additional work for the nursing team and to monitor this possibility on an ongoing basis. Our team met with the nursing team to further understand the workload and perioperative tasks each nurse is responsible for completing. We learned that patients often rely heavily on nurses to ask additional questions and concerns, which are typically made through electronic messages and/or phone calls. Our scheduling process was designed to divert these messages from the nursing team to our chronic pain team. This allowed nursing to be responsible only for verbally discussing the class during the pre-operative visits with the patient and then making sure that the scheduling order was placed. Our team monitored number of patient messages and phone calls as a result of the class and will continue to gather this data to allow for adjustments to the scheduling process to be made as needed.

#### Need for Patient Education on the Use of Online Technology to Access the Class

Our system has experienced an uptick in utilization of virtual technology for healthcare in response to the COVID pandemic. We utilize secure, encrypted technology through the electronic medical record that also allows for group visits. After the initial pilot we recognized a need to provide multiple layers of education prior to and during the group visit to ensure patient engagement and to troubleshoot technology difficulties. Each class was delivered with an administrative staff present; as the instructor focused on delivering the class content, the other staff addressed participant logistic and technologic issues.

#### Operational Challenges in Care Delivery Due to the COVID Pandemic

The COVID pandemic has created specific challenges in surgical care. In particular our workflow was impacted by 1) clinical staff shortages, 2) surgical delays leading to patient uncertainty and distress due to waiting times and 3) conversion to online/virtual delivery of care. Nursing shortages result in provider burnout and clinician overwhelm with integration of a new process or project into their already busy workday. We streamlined the workflow *via* the automated scheduling process to minimize burden on nursing staff. Anecdotally, during the class patients reported increased anxiety associated with care interruption and extended surgical delays, thus underscoring the importance of the class. Observed limitations include difficulty treating patients who reside out-of-state, and, for some patients, apprehensiveness about virtual care.

## Discussion

To our knowledge, this is the first report of pragmatic implementation of an online clinician delivered behavioral intervention targeting spine surgery candidates. We sought to address key gaps identified by the HHS by integrating online-delivered Empowered Relief as standard care for chronic pain and in spine surgery patients at an academic medical center. We described a model of clinical integration based in the RE-AIM framework that dually considered the needs of patient and clinician stakeholders. Our model underscores the importance of developing systems to enhance provider and patient engagement in augmenting standard care with stakeholder education, creation of an efficient clinician-centered workflow and continuous improvement. Our model may serve as an initial implementation blueprint for other healthcare systems wishing to initiate similar initiatives.

A standard care model for behavioral pain medicine stands in stark contrast to current models of behavioral health service delivery wherein typically only patients at highest risk are selected for therapist intervention. Online options offer patients the option of home-based receipt of care with a goal of optimizing the surgical outcomes of all patients rather than treating only those at the greatest risk. Engagement rates for chronic pain (72%) and spine surgery (61%) cohorts suggest good initial feasibility for a standard care model and high rates of patient satisfaction. Further study is needed on larger sample sizes to understand patient perceptions and class utility more broadly.

Previous investigations have revealed that another barrier to behavioral pain care is patient reluctance to see a psychologist (19). Our messaging to the patients and providers is that participation in the class is not only standard of care but “best practice” for multimodal pain management. This universal messaging reduces stigma associated with behavioral treatment and supports a cultural shift of behavioral health integration into chronic pain and surgical care. Given the high rates of engagement in the Empowered Relief classes, our findings support continuation of this model to enhance patients' engagement in behavioral pain care. Engagement rates in the surgical group were somewhat lower than the general chronic pain group. It is possible that the real-time scheduling process for the surgical cohort, could have contributed to lower engagement. While it was designed for convenience, patients may have less of an investment in the class, sense of choice or understanding of the class compared to the chronic pain cohort where scheduling occurs after a visit with a pain psychologist explaining the class in detail.

This report also highlights the unique challenges that exist within the context of the COVID pandemic. There is a need to maintain continuity of care and support patients who are experiencing extended delays to surgery. This is an emergent and understudied issue. A recent systematic review on the impact of the COVID pandemic on surgical practices similarly reported that there are no studies addressing the clinical impact of delaying surgical care during lockdowns (20). Brief, online perioperative pain behavioral intervention may be increasingly important in the context of the pandemic where fewer resources may exist, surgical wait times may be extended by months, and patients may be experiencing compounded stress and pain related to the pandemic and to disrupted medical care.

### Challenges and Limitations

We highlight several challenges and opportunities for improvement. Our findings regarding class acceptability and efficacy were limited by low survey response rates. There are several factors to consider: (1) It is likely that patient habituation to multiple electronic messages prior to surgery contributed to low survey completion; (2) patients were not incentivized to complete the class satisfaction survey; (3) patients receive multiple other patient satisfaction surveys as part of standard of care. Implementing a system that would immediately deploy the anonymous satisfaction survey to class attendees may enhance patient survey completion. Additionally, survey completion rates may be improved with compensation or incentives.

Findings from our project highlight several opportunities for future research. First, there is a need to understand the efficacy of brief, online delivered behavioral intervention in the perioperative period for spine surgery. Opportunities exist to examine the impact of single-session perioperative interventions on surgical outcomes such as pain, pain related coping and opioid use. Research is needed to elucidate the unique medical and psychological challenges of the spine surgery candidate in the context of the COVID pandemic. Such information will inform improvements in processes and systems to support these patients fully and enhance continuity of care.

## Data Availability Statement

The original contributions presented in the study are included in the article/supplementary material, further inquiries can be directed to the corresponding author/s.

## Ethics Statement

This study was reviewed and approved by the Cleveland Clinic Institutional Review Board.

## Author Contributions

SD, JS, AS, and BD contributed to concept and design of the study. SD and AS facilitated data gathering and analysis. SD wrote the first draft of the manuscript. BD heavily edited the manuscript. NT assisted in data analysis. All authors assisted in editing and revision of the final manuscript.

## Conflict of Interest

The authors declare that the research was conducted in the absence of any commercial or financial relationships that could be construed as a potential conflict of interest.

## Publisher's Note

All claims expressed in this article are solely those of the authors and do not necessarily represent those of their affiliated organizations, or those of the publisher, the editors and the reviewers. Any product that may be evaluated in this article, or claim that may be made by its manufacturer, is not guaranteed or endorsed by the publisher.

## Footnotes

^1^Ziadni MS, You DS, Tanner R, Salazar B, Jaros S, Ram J, et al. “My Surgical Success”: Impact of a Digital Behavioral Pain Medicine Intervention on Pain After Orthopedic Trauma Surgery – A Pilot Randomized Controlled Clinical Trial. (under review).
